# Truncated Hemoglobins 1 and 2 Are Implicated in the Modulation of Phosphorus Deficiency-Induced Nitric Oxide Levels in *Chlamydomonas*

**DOI:** 10.3390/cells8090947

**Published:** 2019-08-21

**Authors:** Valentina Filina, Alexandra Grinko, Elena Ermilova

**Affiliations:** Biological Faculty, Saint-Petersburg State University, Universitetskaya nab. 7/9, Saint-Petersburg 199034, Russia

**Keywords:** truncated, hemoglobins, phosphorus deprivation, acclimation, nitric oxide

## Abstract

Truncated hemoglobins (trHbs) form a widely distributed family of proteins found in archaea, bacteria, and eukaryotes. Accumulating evidence suggests that trHbs may be implicated in functions other than oxygen delivery, but these roles are largely unknown. Characterization of the conditions that affect trHb expression and investigation of their regulatory mechanisms will provide a framework for elucidating the functions of these globins. Here, the transcription of *Chlamydomonas* trHb genes (*THB1*–*12*) under conditions of phosphorus (P) deprivation was analyzed. Three *THB* genes, *THB1*, *THB2*, and *THB12* were expressed at the highest level. For the first time, we demonstrate the synthesis of nitric oxide (NO) under P-limiting conditions and the production of NO by cells via a nitrate reductase-independent pathway. To clarify the functions of THB1 and THB2, we generated and analyzed strains in which these THBs were strongly under-expressed by using an artificial microRNA approach. Similar to THB1 knockdown, the depletion of THB2 led to a decrease in cell size and chlorophyll levels. We provide evidence that the knockdown of THB1 or THB2 enhanced NO production under P deprivation. Overall, these results demonstrate that THB1 and THB2 are likely to contribute, at least in part, to acclimation responses in P-deprived *Chlamydomonas*.

## 1. Introduction

Truncated hemoglobins form a widely distributed family of proteins found in archaea, bacteria, and eukaryotes [1]. Truncated hemoglobin sequences are 20–40 amino acid residues shorter than full-length hemoglobins and form a characteristic helix arrangement folded in a 2-on-2 α-helical sandwich [2]. *Chlamydomonas reinhardtii* (*Chlamydomonas* throughout) has 12 truncated hemoglobins named THB1–12 [3,4]. Significant advances have been made in the structural analysis of four THBs, THB1–THB4 [5,6,7,8]. Recent molecular studies have identified several conditions that affect THB expression. For instance, *THB8* transcripts accumulate significantly in hypoxic conditions [3]. Another two THBs, THB1 and THB2, were linked to nitrogen metabolism [5] and sulfur starvation [9]. It has been shown that *THB1* and *THB2* expression is regulated by NIT2, a major transcription factor for nitrate assimilation genes [5,10], and *THB2* is also induced in nitrogen-free medium [11]. Moreover, expression of *THB1* and *THB2* is controlled by nitric oxide [12].

Truncated hemoglobins have also been implicated in nitric oxide (NO) scavenging to protect cells from the accumulation of the radical molecule [13,14]. Recently, THB1 has been determined to modulate NO and thereby control NO-based signaling cascades [10]. Importantly, NO levels increase when the extracellular NO_3_^–^/NH_4_^+^ balance is shifted to ammonium or when sulfur is depleted, and this agrees with the expression patterns of *THB1* [9,12,15]. In higher plants, NO is formed in different stress conditions, including phosphorus (P) deficiency [16,17]. Although NO in *Chlamydomonas* originates from nitrite during nitrogen or sulfur deprivation, the generation of this molecule in P-deprived cells has not been demonstrated in any studies to date.

P, in the form of phosphate (Pi), is an essential nutrient for all photosynthetic organisms, including *Chlamydomonas* [18,19,20]. To cope with Pi limitations, *Chlamydomonas* has evolved mechanisms to improve the acquisition, remobilization, and conservation of Pi [21]. Pi starvation response 1 (PSR1)—a transcription factor in the MYB family—has been identified as a regulator of cell processes under Pi deprivation conditions [22,23,24,25]. In addition to P-specific responses, *Chlamydomonas* cells also exhibit general nutrient stress responses that slow metabolism [19,26]. We hypothesize that similar components might play a role in signaling pathways during acclimation to macronutrient limitation.

In higher plants, an important and ubiquitous signal molecule is NO [27,28,29]. There are a number of studies on the roles of NO in *Chlamydomonas* [3,30,31,32,33]. Recently, a dual system composed of molybdoenzyme nitrate reductase (NR) and nitric oxide-forming nitrite reductase (NOFNiR) has been established, and the complex was found to catalyze the production of NO from nitrite in aerobic conditions [34]. The possibility that *Chlamydomonas* generates NO through NR-independent processes in addition to the nitrite-dependent pathway has also been considered [3,33]. Nevertheless, there remains uncertainty about the mechanism by which NO is formed when the dual system NR–NOFNiR is not functional and the means by which NO concentrations in *Chlamydomonas* are regulated. This prompted us to look for potential THBs that might mediate the levels of NO in macronutrient-deprived cells.

The aims of this study are to (1) investigate the relationship between the acclimation to P starvation and *THB* expression by comparing physiological responses to P limitation in wild-type and *ami*RNA-*THB* strains and (2) elucidate the role of THB1 and THB2 in the regulation of cell acclimation. We found that three THBs are highly induced in P-limiting conditions and that at least two of them, THB1 and THB2, are involved in the acclimation process, apparently by regulating NO levels.

## 2. Materials and Methods

### 2.1. Strains, Growth, and Cell Treatments

The *Chlamydomonas* strains used are given in Supplementary Table S1. Cells were grown in tris-acetate-phosphate (TAP) medium (http://www.chlamy.org/TAP.html) under continuous illumination by white light at 22 °C with constant orbital agitation at 90 rpm. The TAP medium was supplemented with 100 mg/L arginine when required. The strain CC124 used in this work failed to induce NRT2.1 [15] in nitrate, suggesting a nonfunctional NIT2 in this mutant (Supplementary Figure S1).

To induce phosphorus starvation, the cells grown in TAP medium were collected at the midexponential phase of growth by centrifugation (4000× *g*, 5 min), washed twice with phosphorus-free medium (TA), and then incubated in TA. The P-free medium was prepared as reported previously [35]. At each harvesting time, the number of cells was recorded by employing a counting chamber. Four-hundred cells from each sample were scored for three biological replicates. The number of viable cells was counted microscopically with the use of 0.05% (*v*/*v*) Evans blue (DIA-M, Russia) as previously described [36]. The number of non-viable (stained) and viable (unstained) cells were determined. The total chlorophyll content (µg/mL) was quantified according to [37].

### 2.2. RNA Extraction and Quantitative RT-PCR

The total RNA was extracted, and cDNA strands were synthesized as described previously [9]. Real-time quantitative RT-PCR (RT-qPCR) reactions were performed on the Light Cycler Instrument (CFX96 Real-Time PCR Detection System, Bio-Rad) using SYBR Green I following a previously reported protocol [38]. The primer sequences are listed in Supplementary Table S2. The relative gene expression ratios were normalized with *RACK1* (receptor of activated protein kinase C; Cre06.g278222, formerly termed CBLP) using the ΔC_T_ and ΔΔC_T_ methods [38,39]. All reactions were performed in triplicate with at least three biological replicates. Student’s *t*-tests were used for statistical comparisons. *P*-values of <0.05 were considered to be significant.

### 2.3. Generation of THB2-Under-Expressing Strains

The miRNA targeting *Chlamydomonas THB2* was designed as described in [40] using the WMD3 tool at http://wmd3.weigelworld.org. The primers used for plasmid preparation were 5′-ctagtGTGCTCCTCCTTCAATATTTAtctcgctgatcggcaccatgggggtggtggtgatcagcgctaTAAAAATTGAAGGAGGAGCACg-3′ and 5′-ctagcGTGCTCCTCCTTCAATTTTTAtagcgctgatcaccaccacccccatggtgccgatcagcgagaTAAATATTGAAGGAGGAGCACa-3′ (uppercase letters indicate miRNA*/miRNA sequences). The *ami*RNA construct (pChlamiRNA2*THB2*) or the empty vector were transformed into the cell-wall-deficient strain cw15-325 by vortexing with glass beads [41]. The strains were selected on TAP agar without arginine and then screened by RT-qPCR for transformants with decreased *THB2* mRNA content. A transformant generated with the empty vector showed the THB expression patterns similar to parental strain (data not shown).

### 2.4. NO Measurements

Cells (45 µg/mL chlorophyll) were deprived of P for the time indicated and then incubated in the presence of 1 µM 4-amino-5-methylamino-2′7′-difluorofluorescein diacetate dye (DAF-FM DA, Sigma–Aldrich) in the last 15 min. Then, cultures were washed in P-free medium, and intracellular generation of NO was evaluated using a microplate reader CLARIOstar (BMG) using previously reported protocols described [9]. When indicated, 2-(N,N diethylamino)-diazenolate 2-oxide sodium salt (DEA-NONOate, Sigma–Aldrich) was added to the medium to a final concentration of 100 μM, and fluorescence was measured 30 min later. The selective NO scavenger 2-phenyl-4,4,5,5-tetramethylimidazoline-1-oxyl 3-oxide (cPTIO, Sigma–Aldrich, St. Louis, MO, USA) was used in a final concentration of 300 μM. Excitation and emission wavelengths for the NO indicator were 483 ± 14 and 530 ± 30 nm, respectively. Cell autofluorescence was subtracted from the total fluorescence obtained. Fluorescence levels were expressed as arbitrary units (chlorophyll/10^6^ cells). Three technical replicates per condition were included on each plate, and each experiment was performed three times independently.

### 2.5. NO Imaging by Confocal Microscopy

For confocal microscopy imaging, cells were grown and treated as described above. Intracellular production of NO was visualized using the Leica TCS-SP5 confocal microscope (Leica-Microsystems, Germany). The excitation/emission settings were 488/500–544 nm for DAF-FM DA. Chlorophyll fluorescence was captured across a window of 600–680 nm. The images shown were analyzed and processed with the Leica confocal software LAS AF (Leica-Microsystems, Germany) according to [42]. The experiment was performed in triplicate.

## 3. Results

### 3.1. Expression of THBs upon P-Limiting Conditions

The gene *PHOX* encodes the major secreted phosphatase in *Chlamydomonas* [20]. Its low transcription levels in nutrient-replete medium and its strong inducibility in P-free medium qualifies this gene as a marker for studying P limitation-induced responses in *Chlamydomonas* [24]. Using PHOX as a P starvation stress marker, we set out to investigate whether any *THB*s are upregulated in P-deprived cells.

**Figure 1:**
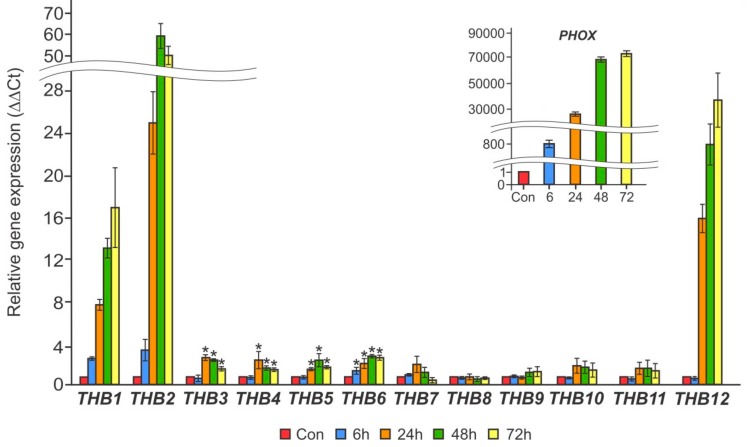
The effects of P starvation on the relative transcript abundance of *THB1–12* genes in the cw15-325 strain. Levels of gene transcripts are calculated as times of relative abundance with respect to the housekeeping control gene (*RACK1*), which has a value of 1. The figure inset shows the *PHOX* transcript accumulation. Data are the means ± SE from three biological and two technical replicates obtained by quantitative RT-PCR. Asterisks indicate statistically significant differences between the untreated and treated P-deprived cells (* *P* < 0.05).

In the first set of experiments, we used cells grown by long-term incubation in TA medium. Quantitative RT-PCR analysis showed that three genes, *THB1*, *THB2*, and *THB12*, were strongly induced after 24 h of P deprivation, and their transcript levels were increased for up to 72 h by 17-, 50-, and 26-fold, respectively (Figure 1). Other upregulated *THB*s (*THB3*, *THB4*, *THB5*, and *THB6*) reached a maximum of 3–3.5-fold induction after 24–72 h of cultivation in P-free medium. Expression of the other *THB* genes either remained constant or declined slightly. It is noteworthy that cw15-325 cells were fully viable after 72 h of P deprivation, which is consistent with previously reported results [43]. These data indicate that at least three members of the THB family may play a role during acclimation to P starvation.

**Figure 2:**
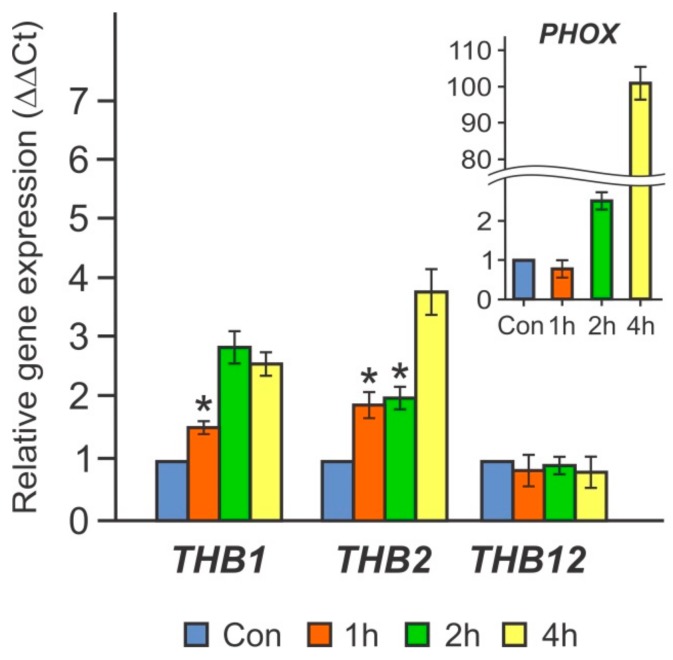
The effects of short-term incubation in phosphorus-free medium (TA) on the relative transcript abundance of *THB1* and *THB2* in the cw15-325 strain. Levels of gene transcripts are calculated as times of relative abundance with respect to the housekeeping control gene (*RACK1*), which has a value of 1. The figure inset shows the *PHOX* transcript accumulation. Data are the means ± SE from three biological and two technical replicates obtained by quantitative RT-PCR. Asterisks indicate the statistically significant differences between the untreated and treated P-deprived cells (* *P* < 0.05).

Interestingly, THB1 and THB2 are truncated hemoglobins whose expression has been found to increase strongly during S deprivation [9]. Moreover, *THB2* was upregulated in N-free medium [10,11]. Therefore, these two THBs were chosen for further analysis. As shown in Figure 2, in cw15-325 cells transferred into TA- medium for 1–4 h, the transcript levels of the genes *THB1* and *THB2* increased by 3-fold and 4-fold, respectively. These results suggest that these two *THB*s may be subject to similar regulatory controls.

### 3.2. NIT2, But Not Nitrate Reductase, Is Required for both THB1 and THB2 Transcription in P-Starved Cells

**Figure 3:**
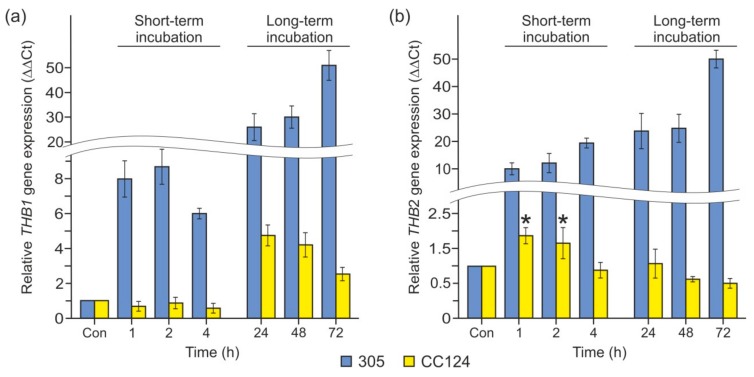
Expression analysis of the *THB1* and *THB2* genes in mutants defective in genes encoding nitrate reductase (NR) and the NIT2 transcription factor during P starvation. (**a**) *THB1* expression levels in the 305 (*nit1*) and CC124 (*nit1nit2*) strains; (**b**) *THB2* expression levels in the 305 (*nit1*) and CC124 (*nit1nit2*) strains. In (**a**,**b**), the levels of gene transcripts are calculated as times of relative abundance with respect to the housekeeping control gene (*RACK1*), which has a value of 1. Data are the means ± SE from three biological and two technical replicates obtained by quantitative RT-PCR.

In *Chlamydomonas*, NR is involved in the regulation of *THB1* and *THB2* expression via the production of NO [6,10]. To test whether NR plays any role in triggering *THB1* and *THB2* transcription under P deprivation, we used the 305 mutant, which is affected in NAD(P)H-NR activity and without diaphorase-NR activity [44]. As shown in Figure 3a, the gene *THB1* exhibited an increase in transcript level in P-starved *nit1* cells compared with P-replete cells. In addition, the levels of the *THB2* transcripts were not diminished in the *nit1* cells (Figure 3b). Therefore, the *nit1* mutation had no effect on the abundance of P deficiency-responsive *THB1* and *THB2* transcripts.

The transcription regulator NIT2 is known to play an important role in controlling the expression of *THB1* and *THB2* [5,10,12]. As demonstrated in Figure 3, the levels of both *THB* mRNAs were impaired in the double mutant CC124 (*nit1nit2*) incubated in TA. However, upregulation of *THB1* and *THB2* in response to P deficiency was partially independent of NIT2. Notably, P depletion slightly (4.8–2.6-fold) increased *THB1* mRNA levels in CC124 after 24–72 h of incubation (Figure 3a). In contrast, the transcript abundance of *THB2* showed a maximum increase of 2-fold after 1–2 h of exposure to P limitation (Figure 3b). Remarkably, *nit1* and *nit2* mutations had no effect on the abundance of P deficiency-responsive *PHOX* transcripts (Supplementary Figure S2).

Taken together, these results strengthen the idea that the expression of *THB1* and *THB2* in P-starved cells is regulated by several factors: NIT2 and one or more unknown transcription activators. These results also indicate that NR is not essential for this control.

### 3.3. NO Is Produced in P-Deprived Cells

**Figure 4:**
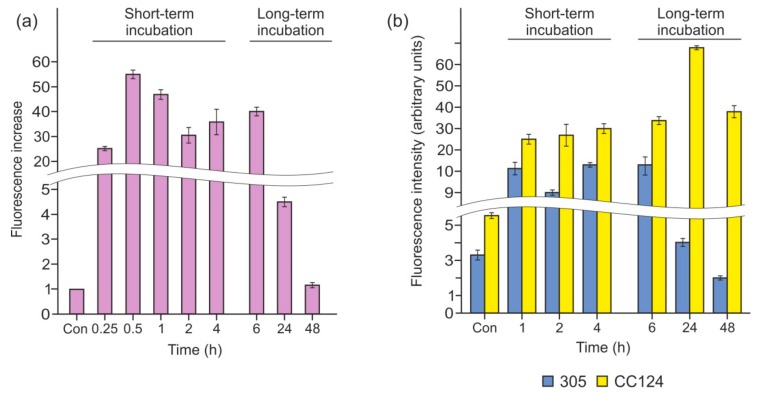
Nitric oxide (NO) generation following the removal of P from the medium. (**a**) Fluorescence increase was measured in the cw15-325 strain following the removal of P from the medium. Fluorescence intensity due to intracellular NO was determined using 1 µM -amino-5-methylamino-2′7′-difluorofluorescein diacetate dye (DAF-FM DA), and fluorescence in tris-acetate-phosphate (TAP) was used as control. The increase in fluorescence at each time point of incubation in P-free medium is shown. Data are the means ± SE from three biological replicates. (**b**) Fluorescence increase was measured in the CC124 and 305 strains following incubation in TA medium. Fluorescence intensity was expressed as arbitrary units per chlorophyll cells 10^−6^. Cell autofluorescence was subtracted from the total fluorescence obtained. In (**a**,**b**), the production of NO was detected by the microplate reader CLARIOstar (BMG).

It has been shown previously that *Chlamydomonas* cells starved of N or S produce NO [9,30,33]. To elucidate whether P limitation triggers NO formation, we examined intracellular NO levels using the microplate reader CLARIOstar (BMG) after incubation with the NO-specific fluorescent probe DAF-FM DA. When cells were transferred into P-free medium, the fluorescence level increased to approximately 25 times that of the control after 15 min and reached a maximum of 55-fold after 30 min exposure to P deprivation (Figure 4a). Remarkably, the NO signal remained at a high level for 1–6 h (~40-fold increase) and then decreased significantly after 24 h of incubation. This result demonstrates that P deprivation stimulates NO production, and NO levels are regulated by cells.

### 3.4. P Deprivation Induces NO Generation without NR–NOFNiR

In *Chlamydomonas*, the dual system composed of NR and NOFNiR is responsible for the reduction of nitrite to NO [34]. However, NO was monitored in cells incubated in ammonium-containing medium (Figure 4a), in which NR activity is not detected. To further confirm the function of another (NR-independent) system in the synthesis of NO, we also tested NR-deficient mutants (Figure 4b). When strains CC124 and 305 were subjected to P starvation, the NO signal was also detected. This finding indicates that P depletion induced NO production not via NR–NOFNiR. Interestingly, the signal gradually decreased in the 305 strain (Figure 4b), and this was concomitant with the upregulation of *THB1* and *THB2* (Figure 3). In contrast, in CC124, P deprivation led to extensive NO formation, even after 24 and 48 h of treatment (Figure 4b). Therefore, reduced amplitudes of *THB1* and *THB2* transcription correlated with increasing levels of NO signal. This correlation represents one line of evidence in support of the idea that hemoglobin 1, 2, or both can oxygenate NO to nitrate in P-deprived cells.

**Figure 5:**
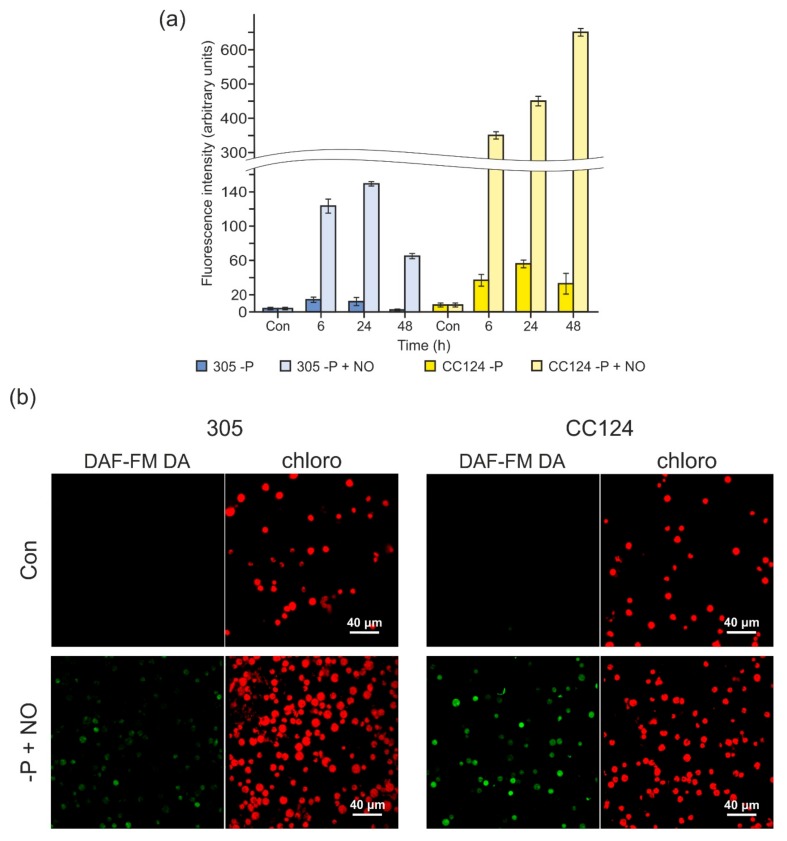
The effects of 2-(N,N diethylamino)-diazenolate 2-oxide sodium salt (DEA-NONOate) on fluorescence intensity in NR-deficient strains upon P deprivation. (**a**) Fluorescence increase by NO treatment (100 µM DEA-NONOate) was detected in two mutants (305 and CC124) after the removal of P from the medium. DAF-FM DA (1 µM) was added in the last 15 min. Fluorescence intensity is expressed as arbitrary units per chlorophyll cells 10^−^^6^. Cell autofluorescence was subtracted from the total fluorescence obtained. The production of NO was measured by the microplate reader CLARIOstar (BMG). Data are the means ± SE from three biological replicates. (**b**) Visualization by confocal microscopy of DEA-NONOate-induced NO generation in the 305 and CC124 strains. The images show the detected cell populations. In each series, the “Con” panels correspond to cells grown in TAP medium, and the “−P+NO” panels correspond to cells subjected to P starvation (24 h) and treated with 100 μM DEA-NONOate (NO). Green indicates the presence of NO, while red fluorescence corresponds to chlorophyll autofluorescence.

Furthermore, the effects of exogenous NO on fluorescence signals were compared in the 305 and CC124 strains. The addition of DAE-NONOate largely increased the NO levels in P-deprived cells (Figure 5a), with the fluorescence in the CC124 strain higher than that in the 305 strain, suggesting that THB1, THB2, or both could be involved in the modulation of NO levels under P-limiting conditions. These results were also confirmed by confocal microscopy data. As shown in Figure 5b, in the presence of DEA-NONOate, the P-starved cells of the 305 strain displayed a weaker green signal than that produced by the CC124 cells.

### 3.5. Knockdown of THB1 or THB2 Results in Increased NO Production

**Figure 6:**
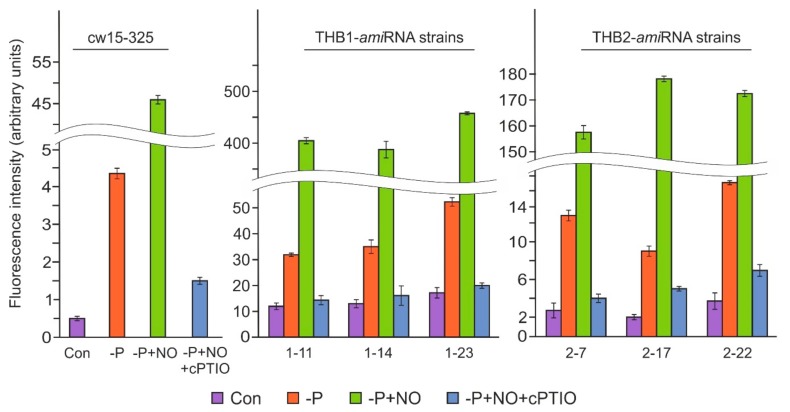
NO generation in *THB2-ami*RNA following the removal of P from the medium. A fluorescence increase was measured in the parental and THB2-under-expressing strains after a 24 h incubation in TA medium with or without 100 µM DEA-NONOate. 300 μM cPTIO was added 5 min before DEA-NONOate. The fluorescence intensity was measured and expressed as described in Figure 5a. Data are the means ± SE from three biological replicates.

In a previous study on *Chlamydomonas*, the under-expression of *THB1* led to an increase in NO production during S starvation [9]. To elucidate the possible role of THB1 in the modulation of NO levels in P-deprived cells, three *THB1-ami*RNA strains [9] were used for further analysis. Weak fluorescence levels were observed in the parental strain and all three transformants before P depletion, with the fluorescence observed in the *THB1*-under-expressing strains higher than that detected in the WT (Figure 6). After 24 h of the P-deficiency treatment, the NO levels in the THB1-knockdown strains were significantly higher than those in the parental strain (7.7–12-fold). Moreover, the NO donor, DEA-NONOate, resulted in an additional increase in the fluorescence level in P-limited cells, with the strongest signal detected in the *THB1*-*ami*RNA strains. These results suggest that THB1 has a role in NO dioxygenation during acclimation to P deprivation.

To understand the relative importance of THB2 in the cellular response to P deprivation, we generated *THB2*-under-expressing strains (*THB2-ami*RNA) using the artificial microRNA approach [40]. RT-qPCR analyses revealed that the transcript levels of *THB2* in the *ami*RNA-*THB2*-7, *ami*RNA-*THB2*-17, and *ami*RNA-*THB2*-22 transformants were 6%, 7%, and 12% of those in the parental cells, respectively (Supplementary Figure S3a). Interestingly, similar to the *THB1*-under-expressing strains, the *ami*RNA-*THB2*-7, *ami*RNA-*THB2*-17, and *ami*RNA-*THB2*-22 cells had a smaller average diameter (7.3 ± 0.6 µm) compared with the WT (8.5 ± 0.4 µm). Furthermore, levels of chlorophyll were reduced to 42–55% of WT content (Supplementary Figure S3b). However, the downregulation of THB2 did not affect the viability of P-deprived cells (Supplementary Figure S3c).

Next, we examined the NO levels in *ami*RNA-*THB2*-7, *ami*RNA-*THB2*-17, and *ami*RNA-*THB2*-22 after 24 h of P deprivation (Figure 6). In the *THB2*-knockdown cells starved of P, the fluorescence level was higher than that in the WT cells. When the *THB2-ami*RNA strains were exposed to DEA-NONOate, the DAF-FM DA signal increased strongly compared with untreated P-deprived cells. However, the observed NO levels were significantly lower than those in the DEA-NONOate-treated *THB1-ami*RNA cells (Figure 6). Notably, DEA-NONOate had no effect after preincubation of all strains with cPTIO. Thus, these results suggest that THB2 may play a role in the regulation of NO levels during P starvation.

## 4. Discussion

Distantly related to vertebrate hemoglobins, the superfamily of trHbs is present in various pro- and eukaryotes with biological roles other than oxygen delivery [2,45]. The unicellular alga *Chlamydomonas*, which has 12 nuclear *THB*s, is an excellent model organism to study variations in the expression mode and functions among the members of this superfamily [3]. In this work, we report original insights into the expression of *THB*s in response to P deprivation.

In *Chlamydomonas*, the transcription of *THB*s is influenced by several factors, including nitrogen and sulfur deprivation [5,9,11]. Because P is third among the major macronutrients involved in cell growth, we tested whether P limitation also changed *THB* expression in this microalga. This study shows that depletion of P resulted in the upregulation of a suite of *THB* genes (Figure 1), particularly *THB1*, *THB2*, and *THB12*, which were expressed at a high level. Interestingly, these three *THB* genes are highly inducible in S-deprived cells [9]. However, the expression patterns of the *THB1*, *THB2*, and *THB12* genes differed between the two nutrient-stress conditions: the accumulation of the analyzed mRNAs occurred more slowly and with a reduced amplitude in cells exposed to P starvation compared with cells subjected to S deprivation (Figure 1 and Figure 2) [9]. This observation is consistent with the fact that it takes longer for *Chlamydomonas* to respond to P limitation than to S limitation [23,26].

Two *THB* family members, *THB1* and *THB2*, were previously shown to be regulated by the transcription factor NIT2 [5,10,12]. Although NIT2 is also required for the expression of these genes during P limitation, one or more unknown transcription activators operating in P-deprived *Chlamydomonas* appeared to control the transcription of both genes (Figure 3). Thus, the identification of additional transcription activators for *THB1* and *THB2* expression is an important subject to further study.

Moreover, NO regulates *THB1* and *THB2* expression [12]. As mentioned above, the dual system NR–NOFNiR is responsible for NO production in *Chlamydomonas* [34]. In this study, we demonstrated that NO was synthesized under P-limiting conditions (Figure 4a). However, the accumulation of *THB1* and *THB2* mRNAs was not impaired in *nit1* mutant cells during P deprivation (Figure 3). This finding supports the scenario of NR-independent NO production. Our investigation confirms that NR-defective mutants generated NO in P-depleted cells (Figure 4b), which is consistent with previous observations that other (NR–NOFNiR-independent) NO-forming systems function in *Chlamydomonas* [3,33,46]. The mechanism by which P depletion leads to the generation of NO in an ammonium-containing medium in this scenario still needs to be addressed.

In addition, we note that, although both the CC124 and 305 strains generated NO, its levels in the two mutants were regulated differently. In contrast to 305, in the CC124 cells, NO production remained at a high level after 24 and 48 h of P limitation (Figure 4b), suggesting a correlation between the expression levels of *THB1* and *THB2* and the amount of NO. An interesting observation is that DEA-NONOate treatment resulted in stronger NO accumulation in CC124 compared with 305 (Figure 5), indicating that the first strain may have NO-scavenging systems with decreased efficiency.

Here, we provide evidence suggesting that two truncated hemoglobins have a role in the modulation of NO levels upon P deprivation. P depletion led to a more dramatic increase in the DAF-FM DA signal in *THB1*-knockdown strains compared with the WT (Figure 6). This is similar to the stimulatory effects of THB1 reduction on the amounts of NO reported for S- and N-limiting conditions [9,10]. The observed effects might be explained by the fact that THB1 is capable of NO deoxygenation [5,10]. More generally, we could consider that THB1 plays a regulatory role in the control of NO production dynamics to ensure the efficiency of NO scavenging when cells are limited for macronutrients and it could be a part a general response to stress.

We also examined the possible effect of THB2 on NO concentrations during P starvation. Elevated NO levels were detected in *THB2*-under-expressing strains (Figure 6). Notably, compared with the knockdown of THB1, the knockdown of THB2 resulted in a less drastic increase in the fluorescence level. This difference between the *THB1-ami*RNA and *THB2-ami*RNA strains could be explained by some specific physicochemical properties of each of these hemoglobins [6]. It should be noted, however, that the cell size and chlorophyll contents of *THB2*-knockdown cells were slightly reduced relative to the WT and similar to the *THB1*-knockdown cells (Supplementary Figure S2) [9]. This finding supports the hypothesis that the induction of the *THB1* and *THB2* genes is likely to contribute, at least in part, to acclimation responses in S- and P-deprived cells. However, it remains possible that other THBs might also be essential for *Chlamydomonas* responses to macronutrient limitation.

In conclusion, the characterization of *THB* expression and NO production in P-depleted cells expands our understanding of the P deficiency-induced network beyond previous findings of the core components of the regulatory and metabolic responses associated with P deprivation in *Chlamydomonas* [19,20,21,22,23,24,25,26]. The implication of two or even more truncated hemoglobins in NO homeostasis suggests the existence of a molecular mechanism that coordinates all THBs to achieve a balance between NO signaling and NO scavenging upon P-limiting conditions.

## Supplementary Materials

The following are available online at https://www.mdpi.com/2073-4409/8/9/947/s1, Figure S1: Expression analysis of the NRT2.1 gene in strains 305 and CC124; Figure S2: Expression analysis of the PHOX gene in nitrate reductase-deficient mutants during P starvation; Figure S3: Characterization of amiRNA-THB2 strains; Table S1: Chlamydomonas strains used; Table S2: Primers for RT-qPCR analysis.
