# Supplementary material for: Truncated Hemoglobins 1 and 2 Are Implicated in the Modulation of Phosphorus Deficiency-Induced Nitric Oxide Levels in Chlamydomonas
Source: Cells. 2019 Aug 21;8(9):947. doi: 10.3390/cells8090947 (PMC6770159; doi:10.3390/cells8090947)
Supplement: Supplementary file 1 [file cells-08-00947-s001.pdf]

*Article – Supplementary Files*

# Truncated hemoglobins 1 and 2 are implicated in the modulation of phosphorus deficiency-induced nitric oxide levels in *Chlamydomonas*

**Valentina Filina, Alexandra Grinko and Elena Ermilova**

Biological Faculty, Saint-Petersburg State University, Universitetskaya nab. 7/9, Saint-Petersburg 199034, Russia; filina.valya@yandex.ru (V.F); st047636@student.spbu.ru (A.G.)

* Correspondence: e.ermilova@spbu.ru

**Supplementary Figures 1, 2 and 3:**

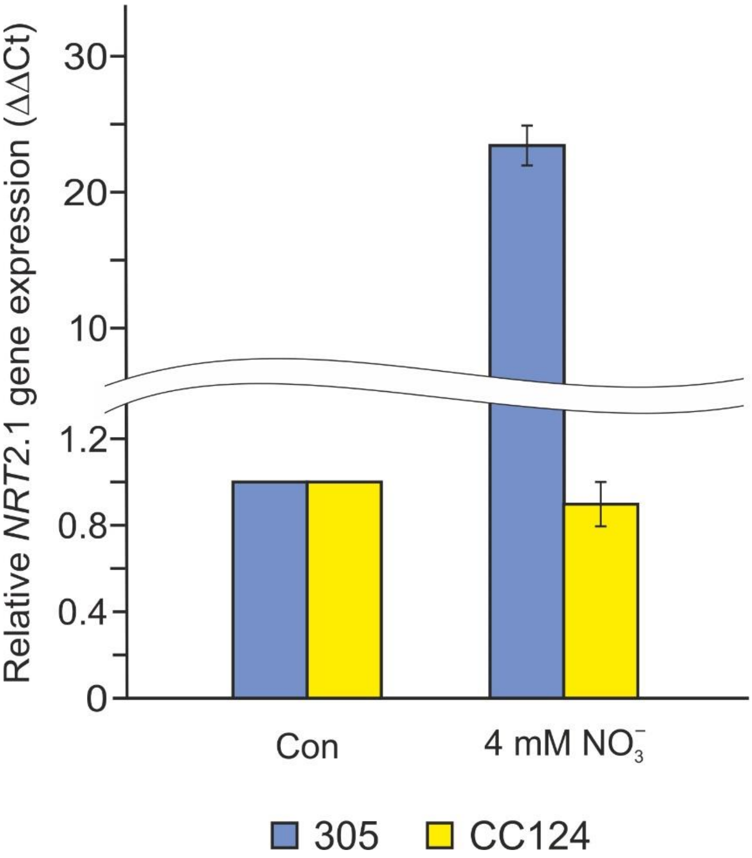


**Figure S1**. Expression analysis of the *NRT2.1* gene in strains 305 and CC124. Cells were grown in ammonium-containing medium (Con) and then transferred to a medium containing 4 mM $KNO_3$ for 1 h. Levels of gene transcripts are calculated as times of relative abundance with respect to the housekeeping control gene (*RACK1*) that has a value of 1. Data are the means ± SE from three biological and two technical replicates obtained by quantitative RT-PCR. Con

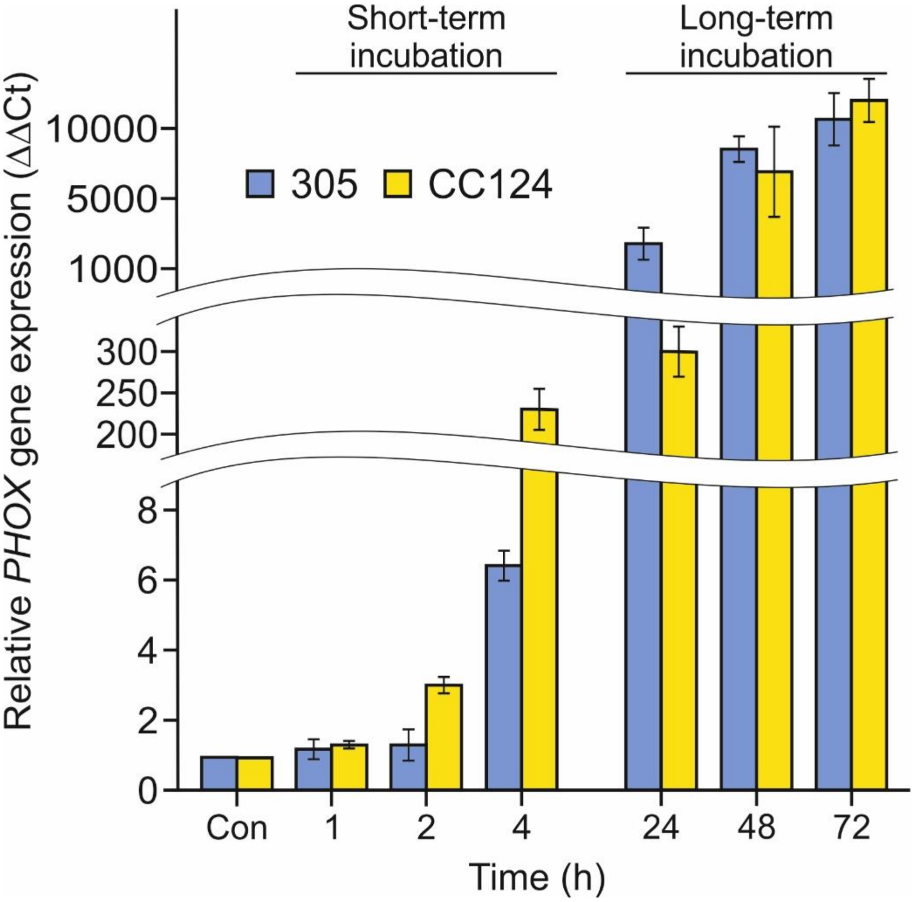


**Figure S2**. Expression analysis of the *PHOX* gene in nitrate reductase deficient mutants during P starvation. Levels of gene transcripts are calculated as times of relative abundance with respect to the housekeeping control gene (*RACK1*) that has a value of 1. Data are the means ± SE from three biological and two technical replicates obtained by quantitative RT-PCR.

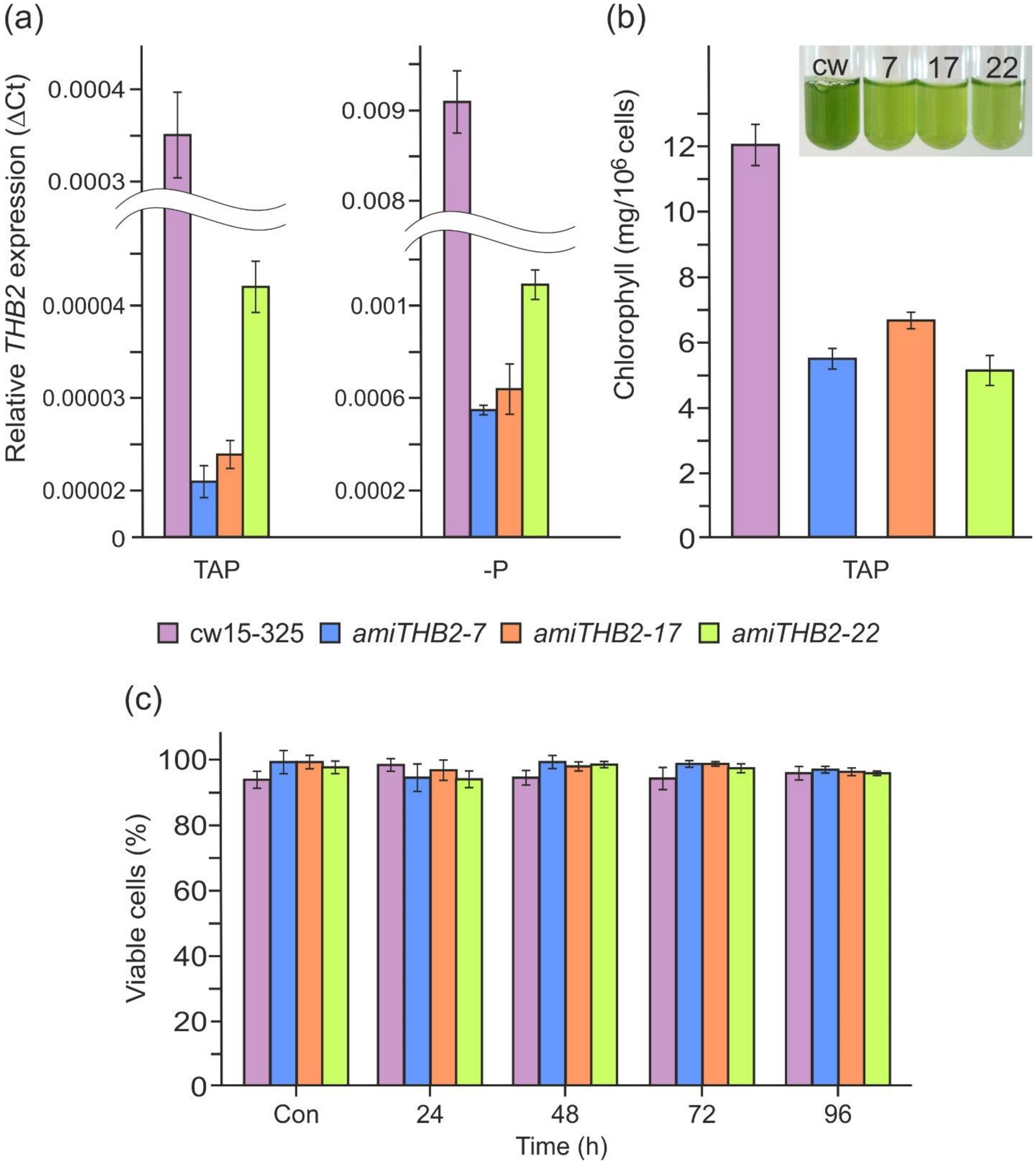


**Figure S3**. Characterization of *amiRNA-THB2* strains: **(a).** Expression analysis of the *THB2* gene in cw15-325 and *ami*RNA-*THB2* cells grown in TAP or incubated in P-free medium for 24 h. Relative expression levels were normalized with the gene expression of *RACK1* and calculated using $\Delta C_T$; all measurements were done in triplicate; **(b)** Comparative chlorophyll contents of parental strain cw15-325 and *ami*RNA-*THB2* strains. Vegetative cells were grown in TAP medium. Insert shows test tubes with the same cell density of cultures (2 $10^6$ cells/ml) in TAP; **(c)** Viability of parental strain cw15-325 and *ami*RNA-*THB2* strains. Vegetative cells were grown in TAP medium and transferred to TA medium for 24h, 48h, 72h or 96h. A viability dye was used to distinguish viable from nonviable cells as explained in the Materials and Methods section. Values are means ± SD (n = 3).

**Table S1**: *Chlamydomonas* strains used

| **Strain** | **Genotype** | **References** |
|---|---|---|
| cw15-325 | *mt+, cw15, arg7* | [37] |
| CC124 | *mt⁻, nit1, nit2* | [37] |
| 305 | *mt⁻nit1* | [44] |
| *ami*THB1-11 | *mt+, cw15* | [9] |
| *ami*THB1-14 | *mt+, cw15* | [9] |
| *ami*THB1-23 | *mt+, cw15* | [9] |
| *ami*THB2-7 | *mt+, cw15* | This work |
| *ami*THB2-17 | *mt+, cw15* | This work. |
| *ami*THB2-22 | *mt+, cw15* | This work |

**Table S2**. Primers for RT-qPCR analysis

| Target gene/Accession number | Primer name | Sequence (5′–3′) | Reference |
|---|---|---|---|
| *THB1*/Cre14.g615400 | *THB1*F<br>*THB1*R | ATGAAGAAGCAGCGCCGCAAAC<br>ACCAGGTCAAAGTGGTGGTGGTTC | [10] |
| *THB2*/Cre14.g615350 | *THB2*F<br>*THB2*R | GCCGGTTGATCCGCGACAAG<br>CGATCCAACTTTTACACCCGCTCAA | [10] |
| *THB3*/Cre04.g218800 | *THB3*F<br>*THB3*R | TCACTTATCGCCAGTCTAGAGGAC<br>CGCTCAGGATGTCGTCTATAAGC | [10] |
| *THB4*/Cre04.g218750 | *THB4*F<br>*THB4*R | GCTTCAAGGAGACGGTGTGAAGTCTAC<br>ACATCCACCGTTGCTGCCACA | [10] |
| *THB5*/Cre07.g351100 | *THB5*F<br>*THB5*R | GGCGTTTTATCGCAAGTTGT<br>CTTGAACGTATCCAGCAGCA | [9] |
| *THB6*/Cre16.g654250 | *THB6*F<br>*THB6*R | CCTGGACTCGATAGCAGAGG<br>TGTCGTGAGAGACGGAACTG | [9] |
| *THB7*/Cre16.g661000 | *THB7*F<br>*THB7*R | CATGGTGCCGTGCTCGTACA<br>CGACCAGCACTGCCTACTTG | [4] |
| *THB8*/Cre16.g661200 | *THB8*F<br>*THB8*R | CGGGAGTCAGCAAGCTGTCAAC<br>CCGCCCGTACACAAACAAGCAC | [4] |
| *THB9*/Cre16.g661250 | *THB9*F<br>*THB9*R | GCTCTCTCTGGTTTTGAAGCAT<br>AGCTGCTCATCTGCGTACAAT | [9] |
| *THB10*/Cre16.g661300 | *THB10*F<br>*THB10*R | TGCTGCGGAGGTGTTCCTTG<br>CATTGCCGCCTCTGCTGATG | [4] |
| *THB11*/Cre16.g662750 | *THB11*F<br>*THB11*R | TTGCGTGCGTCCATGCTGTC<br>CCGGTTGCGGATACACCTCT | [4] |
| *THB12*/Cre16.g663000 | *THB12*F<br>*THB12*R | GACCCCTCACTCATAAAGTTCCT<br>AAGTACTTCATGCCCAGATCAAA | [9] |
| *RACK1*/Cre06.g278222 | *RACK1*F<br>*RACK1*R | CTTCTCGCCCATGACCAC<br>CCCACCAGGTTGTTCTTCAG | [38] |
| *PHOX*/Cre04.g216700 | *PHOX*F<br>*PHOX*R | TTCCGTTTCCGTTCTCTGAC<br>CCCTGCATCTTGTTCTCCAG | [24] |
| *NRT2.1*/Cre09.g410850 | *NRT2.1F*<br>*NRT2.1R* | CGCCGTGGCAACTGACCCTGAG<br>CGCCACCTCCTCCGCACTCCAC | [15] |

Sequences were obtained from Phytozome 12, *Chlamydomonas reinhardtii v5.5*
